# Leber’s hereditary optic neuropathy companied with multiple-related diseases

**DOI:** 10.3389/fnhum.2022.964550

**Published:** 2022-11-03

**Authors:** Ming-ming Sun, Huan-fen Zhou, Qiao Sun, Hong-en Li, Hong-juan Liu, Hong-lu Song, Mo Yang, Da Teng, Shi-hui Wei, Quan-gang Xu

**Affiliations:** ^1^Senior Department of Ophthalmology, The Third Medical Center of PLA General Hospital and Chinese PLA Medical School, Beijing, China; ^2^Senior Department of Ophthalmology, The First Medical Center of PLA General Hospital and Chinese PLA Medical School, Beijing, China; ^3^Department of Ophthalmology, Shanghai Aier Eye Hospital, Shanghai, China; ^4^Beijing Ophthalmology and Visual Sciences Key Laboratory, Beijing Tongren Eye Center, Beijing Tongren Hospital, Capital Medical University, Beijing, China; ^5^Department of Neuro-Ophthalmology, Eye Hospital, China Academy of Chinese Medical Sciences, Beijing, China; ^6^Department of Ophthalmology, Beijing Tiantan Hospital, Beijing, China

**Keywords:** Leber’s hereditary optic neuropathy, optic neuritis, aquaporin-4 antibody, myelin oligodendrocyte glycoprotein antibody, multiple sclerosis

## Abstract

**Objective:**

To elucidate the clinical, radiologic characteristics of Leber’s hereditary optic neuropathy (LHON) associated with the other diseases.

**Materials and methods:**

Clinical data were retrospectively collected from hospitalized patients with LHON associated with the other diseases at the Neuro-Ophthalmology Department at the Chinese People’s Liberation Army General Hospital (PLAGH) from December 2014 to October 2018.

**Results:**

A total of 13 patients, 24 eyes (10 men and 3 women; mean age, 30.69 ± 12.76 years) with LHON mitochondrial DNA (mtDNA) mutations, were included in the cohort. 14502(5)11778(4)11778 &11696(1)12811(1)11696(1)3460(1). One patient was positive for aquaporin-4 antibody (AQP4-Ab), and two were positive for myelin oligodendrocyte glycoprotein antibody (MOG-Ab). Three patients were associated with idiopathic optic neuritis (ON). Two patients were with compression optic neuropathy. Three patients were with the central nervous system (CNS) diseases. One patient was with proliferative diabetic retinopathy (PDR) and one with idiopathic orbital inflammatory syndrome (IOIS). At the onset, visual acuity (VA) in eighteen eyes was below 0.1, one eye was 0.5, five eyes were above 0.5, while VA in sixteen eyes was below a 0.1 outcome, three eyes experienced moderate vision loss. MRI images showed T2 lesions and enhancement in nine patients who received corticosteroids treatment; additional immune modulators treatment was performed on two patients. None of the patients had relapse during the follow-up time.

**Conclusion:**

Leber’s hereditary optic neuropathy can be accompanied with multiple-related diseases, especially different subtypes of ON, which were also exhibited with IOIS and compression optic neuropathy for the first time in this cohort. This condition may be a distinct entity with an unusual clinical and therapeutic profile.

## Introduction

Leber’s hereditary optic neuropathy (LHON) is an inherited optic neuropathy characterized by subacute painless vision loss (Hudson et al., 2008). Retinal ganglion cells (RGCs) are preferentially affected, leading to optic nerve degeneration, but additional extraocular abnormalities have been described in LHON pedigrees. Previous studies suggest that LHON may be a systemic disorder with manifestations in organs other than the optic nerves (Nikoskelainen et al., 1995). These include non-neurological as well as neurological abnormalities. Since LHON was first described in 1926 by Mauksch and his colleagues; the association between CNS inflammatory demyelination and LHON was always controversial (Dujmovic et al., 2016). Until now, the relationship between LHON and the other diseases has not been clear, and whether the other organs may be involved in patients with LHON is unknown.

In clinical, we noticed 13 patients who carried mitochondrial DNA (mtDNA) mutations that were accompanied with the other diseases, including ocular disorders and systemic diseases. We conducted the current study to describe the clinical, laboratory, imaging presentations of MRI, and associated diseases at a single center, using data collected in a retrospective fashion.

## Patients and methods

The clinical records from December 2014 to October 2018 of hospitalized patients carried mtDNA mutations were selected from the Chinese People’s Liberation Army General Hospital (PLAGH) in this study. The study protocol was approved by the institutional review board of the PLAGH and performed in accordance with the content of the Helsinki Declaration. All patients provided written informed consent to undergo examinations.

### Ophthalmologic and associated examinations

Ophthalmological examinations included a test for relative afferent pupillary defect (RAPD), and a direct and indirect ophthalmoscopy to examine the retina. The best corrected visual acuity (BCVA) was tested using a Snellen chart, and a VA below 0.01 was documented with count finger (CF), hand motion (HM), perceived light, and no perceived light. The standard protocol for the Optic Disc Cube 200 × 200 circle scan and the Macular Cube 512 × 128 scan was performed using one of the two spectral-domain optic coherence tomography (SD-OCT) devices (Carl Zeiss Meditec, USA or Heidelberg Engineering, Germany). Blood was collected at the Rheumatologic Research Center in the PLAGH. Cerebrospinal fluid (CSF) samples were collected for the routine testing of white cell counts, total protein levels, and concentrations of IgG. Patients with LHON were diagnosed by screening for mtDNA. Cell-based assays (CBA) to detect the serum aquaporin-4 antibody (AQP4-Ab) and the myelin oligodendrocyte glycoprotein antibody (MOG-Ab) were performed for the patients with ON.

Magnetic resonance imaging was performed on a 3T system in eight patients with a T2-weighted image (T2WI) and a T1-weighted image (T1WI) sequences with fat suppression, and a gadolinium-enhanced T1. Proton Magnetic Resonance Spectroscopy (H-MRS) is performed when patients are accompanied with intracranial lesions.

The following data were analyzed: age, sex, age at the onset, the involved eye, time between two eyes, family history, laboratory findings, associated diseases, treatment, and prognosis.

## Results

### Demographics characteristics

Table 1 summarizes their clinical characteristics, genotypes, and plus diseases. A total of 13 patients (10 males, 3 females; age range, 13–53 years) with genetically confirmed carried mtDNA mutations that were associated with the other diseases. Four patients carried the 11778 mutations, five patients carried the 14502 mutations, one patient carried the 12811 mutations, the 3460 mutations, the 11696 mutations, respectively. And, in one patient, the 11778 mutations were associated with 11696. Three patients had concomitant ION. Two patients complained with MOG-Ab positive; the titers were 1:10 and 1:100, respectively. One patient complained with AQP4-Ab positive. Two patients were with compression optic neuropathy (hypophysoma and intracranial germinoma). Three patients with the central nervous system (CNS) diseases [multiple sclerosis (MS), leigh, and cranial neuropathy]. One patient with PDR and one with idiopathic orbital inflammatory syndrome (IOIS, orbital pseudotumor). Five patients were diagnosed with LHON prior, while eight patients were diagnosed with the other diseases prior. Two patients were confirmed in the maternal family.

**TABLE 1:** Clinical features data for 13 patients.

Case	Age at onset (years)	Involved eye(s)	Time between two eyes	Plus	Mutation	Family history	Visual acuity at onset		VA (outcome)	
							OD	OS	OD	OS
1/M/27	27	OU	/	MOG-ON	12811	/	0.6	0.6	1	0.8
2/M/23	22	OU	1 month	MOG-ON	14502	/	0.6	0.5	0.6	0.15
3/F/45	45	OU	/	NMO-ON	14502	/	CF	CF	CF	CF
4/M/27	26	OU	1 month	ION	11778	/	0.04	CF	0.08	0.02
5/M/17	17	OD	/	ION	11778	Positive	/	CF	/	CF
6/M/29	29	OU	/	ION	11696	/	0.8	0.04	0.8	0.5
7/M/33	33	OS	/	IGM	14502	/	/	HM	/	LP
8/M/19	19	OU	/	MS	14502	/	CF	CF	0.02	0.2
9/F/50	49	OU	6 month	IOIS	14502	/	0.8	0.02	1	0.02
10/M/23	23	OU	/	Hypophysoma	3460	/	CF	0.1	CF	0.1
11/F/13	7	OU	6 years	Leigh	11778	Positive	CF	CF	CF	CF
12/M/53	20	OU	/	Cranial neuropathy	11778	/	CF	CF	CF	CF
13/M/40	20	OU	3 month	PDR	11778, 11696	/	0.04	0.01	0.07	0.02

F, female; M, male; OD, oculus dexter; OS, oculus sinister; OU, oculus uterque; CF, counting fingers; HM, hand motion; ION, idiopathic optic neuritis; IGM, intracranial germinoma; MS, multiple sclerosis; IOIS, idiopathic orbital inflammatory syndrome; PDR, proliferative diabetic retinopathy.

### Clinical characteristics

A total of 182 patients (345 eyes) were diagnosed with LHON in our center, while 13 patients were diagnosed with LHON Plus; the frequency of LHON Plus in LHON was 7.14%. The age onset of LHON with other diseases and pure LHON were (25.9 ± 3.1) and (22.1 ± 0.9) years, respectively. In LHON Plus, 11 patients suffered simultaneous or sequential binocular involved, the time between two eyes from 1 month to 6 years; the average time was (30 ± 17.16) days. There was no significant difference between the age onset, involved eyes, and the time between two eyes of LHON Plus and pure LHON. The BCVA was significant difference between two groups; the visual function was better in LHON Plus than pure LHON, and we suspected the reason was the plus disease accepted effective treatment. The data between the two groups are listed in Table 2.

**TABLE 2:** The clinical characteristics between Leber’s hereditary optic neuropathy (LHON) with other diseases and pure LHON.

	LHON plus	LHON	*P*
The age-onset (y)	25.9 ± 3.1	22.1 ± 0.9	0.28
Involved eyes (binocular)	11	163	0.64
The time between two eyes (d)	30 ± 17.16	48.26 ± 12.74	0.71
BCVA visual acuity	0.51 ± 0.13	0.90 ± 0.03	0.002

BCVA, best corrected visual acuity.

**FIGURE 1:**
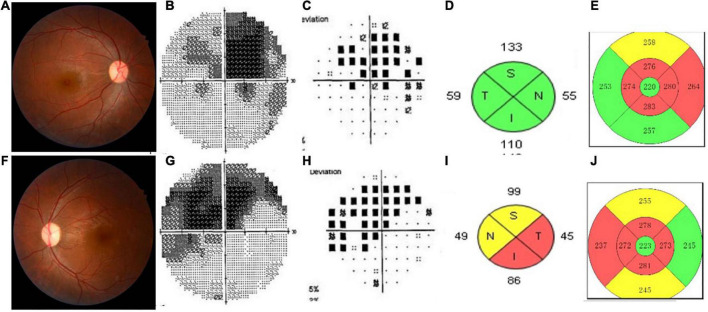
This shows the images evidence and changes in the patients with Leber’s hereditary optic neuropathy (LHON). Color fundus photographs **(A,F)** demonstrate temporal optic nerve pallor OS **(F)**. Humphrey visual field (VF) testing demonstrates a dense superior central scotoma OD **(B,C)** and a central scotoma with a superior arcuate defect OS **(G,H)**, having mean deviations (MD) of –13.54 and –12.73, respectively. SD-OCT imaging of the peripapillary nerve fiber layer (pRNFL) **(D,I)** showed diffuse thinning of the left eye **(I)**. Spectral-domain optic coherence tomography SD-OCT imaging of the macular inner limiting membraneretinal pigment epithelium (ILM-RPE) **(E,J)** revealed ganglion cell atrophy, more severe in the nasal quadrants involved left eye **(J)** than the right eye **(E)**.

Four patients were RAPD positive. Approximately, 76.92% (10/13) patients had pale fundus, especially the temporal side. One patient had edema disk. Two patients had normal fundus. Nine patients (14 eyes) had distinctive visual field (VF) defection, which showed central or eccentric scotoma especially. The results of the VEP examination in 10 patients showed the delayed implicit period and lower amplitude. SD-OCT imaging of the peripapillary nerve fiber layer (pRNFL) showed diffuse thinning in eight patients (14 eyes); SD-OCT imaging of the macular retinal ganglion cell and inner plexiform layers (RGC-IPL) revealed ganglion cell atrophy, more severe in the nasal quadrants. Figure 1 shows the images evidence and changes by fundus photography, humphrey VF testing, and SD-OCT images in different patients.

### Magnetic resonance imaging manifestation and cerebrospinal fluid tests

**FIGURE 2:**
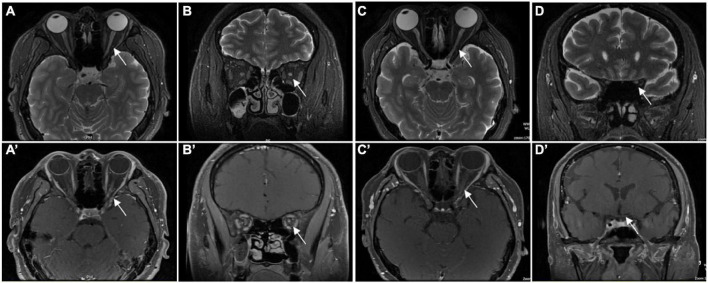
Orbital MRI images in patients who were diagnosed with ON. Panels **(A,C)** (axial) and **(B,D)** (coronal) showed the high signal of the left ON (the white arrow) in T2WI. Panels **(C)** (axial) and **(D)** (coronal) showed the enhancement of left ON (the white arrow) in gadolinium-enhanced T1. Panels **(A′)** (axial) and **(B′)**(coronal) showed the enhancement of left ON (white arrow) in gadolinium enhanced T1. Panels **(C′)** (axial) and **(D′)** (coronal) showed the normal signal of left ON (the white arrow) in gadolinium-enhanced T1.

**FIGURE 3:**
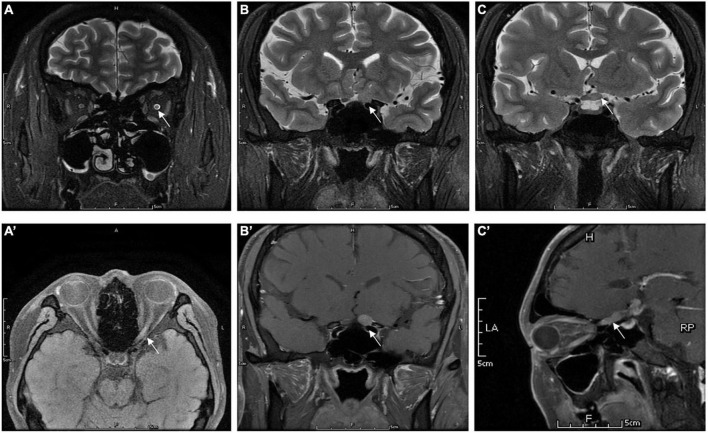
Orbital MRI images in patients who were diagnosed with IGM (case 7): panels **(A)** (axial), and **(B,C)** (coronal) showed the hypersignal of the optic nerve (the white arrow) extends to optic chiasm in T2WI. Panels **(A′)** (axial), **(B′)** (coronal), and **(C′)** (sagittal) showed the enhancement of left ON (the white arrow) in gadolinium-enhanced T1.

**FIGURE 4:**
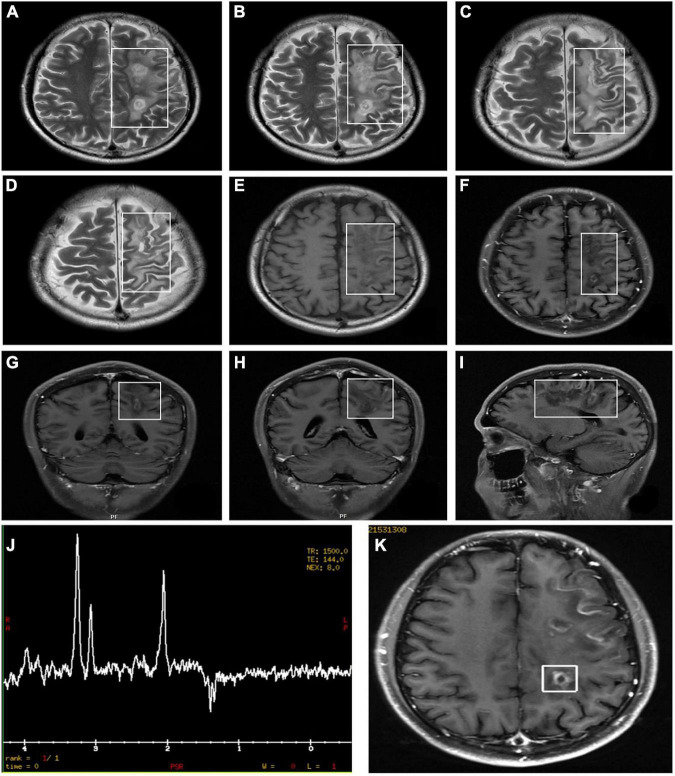
Orbital MRI images in patients who were diagnosed with LHON-MS (case 9). The figure showed multiple lesions presented with an abnormal signal **(A–I)**. MRI findings **(K)** and Proton Magnetic Resonance Spectroscopy (H-MRS) findings **(J)** in the proband of Pedigree 1—multiple demyelinating lesions in the left cerebrum (MRI) and an inverted double peak of lactic acid (Lac) at 1.33 ppm (H-MRS).

Table 3 gives the orbit MRI and CSF test results. The 12 of 13 patients had optic nerve MRI scans in our hospital. T2WI images showed an increased signal of the optic nerve, and post-contrast T1WI with fat suppression showed abnormal enhancement of the optic nerve in six patients (Figure 2). Every segment of the optic nerve may be involved. In the patients with optic nerve tumor, T2WI images showed an increased signal, and post-contrast T1WI with fat suppression showed abnormal enhancement of the enlarged optic nerve (Figure 3). The typical MRI characteristics of MS are as follows: periventricular lesions, which are arranged perpendicular to the ventricular margin, Dawson’s fingers (Figure 4). Axial and coronal T1WI with fat suppression and contrast showed left optic nerve enhancement at the level of the orbital apex, which diagnosed IOIS. T2WI images showed intracranial lesions without enhancement in four patients. H-MRS images showed lactic acid peak in one patient (Figure 4). Seven (7/13, 53.85%) of the patients had lumbar puncture and the CSF test; two had both elevated total protein concentration of 521.5and 527.9 mg/l (normal range, 150–400 mg/l) and elevated IgG concentration of 3.47 mg/dl and 5.33 mg/dl in CSF (normal range, 0–3.4 mg/l, Table 3), respectively.

**TABLE 3:** The characteristics of MRI, treatment, and cerebrospinal fluid (CSF) with Leber’s hereditary optic neuropathy (LHON) plus patients.

Case	MRI	Methylprednisolone, mg, initial		Prevention	CSF		
		Intravenous	Oral		WBC (10^6^/L)	Total protein (mg/L)	IgG (mg/dl)
*N*					–	150∼400	0∼3.4
1/M/27	T2 lesions and enhancement (OS, Orbital) + T2 lesions (intracranial)	500	48	/	/	/	/
2/M/23	T2 lesions and enhancement (Optic chiasm)	1,000	48	Azathioprine	1	341.7	2.51
3/F/45	T2 lesions (Optic chiasm)	1,000	48	Azathioprine/rituximab	0	321.1	2.79
4/M/27	T2 lesions and enhancement (OS, Orbital)	1,000	48	/	2	521.5	3.47
5/M/17	T2 lesions and enhancement (OS, Orbital + Canalicular)	1,000	48	/	2	382.1	2.74
6/M/29	T2 lesions and enhancement (OU, Orbital)	1,000	64	/	0	323.7	2.58
7/M/33	T2 lesions and enhancement (OS, Optic nerve + Optic chiasm)	1,000	64	/	12	527.9	5.33
8/M/19	T2 lesions (intracranial + Optic nerve + Optic chiasm)	250	48	/	1	292.2	1.04
9/F/50	T2 lesions and enhancement (OS, Orbital + Canalicular)	1,000	48	/	/	/	/
10/M/23	Postoperative changes of hypophysoma	/	/	/	/	/	/
11/F/13	T2 lesions (intracranial)	/	/	/	/	/	/
12/M/53	T2 lesions (intracranial)	/	/	/	/	/	/
13/M/40	/	/	/	/	/	/	/

OD, oculus dexter; OS, oculus sinister; OU, oculus uterque.

### Treatment and prognosis

In this study cohort, 75% (18/24) of the eyes experienced severe vision loss (≤0.1) at the onset, only one eye experienced moderate vision loss (0.1–0.5), five eyes experienced mild vision loss (≥0.5). Steroid therapy was performed for nine patients with MRI changes. Two patients received immune modification therapy (azathioprine/rituximab) with AQP-Ab or MOG-Ab positive. Vision improved in seven patients, unchanged in four patients, and worse in two patients (Table 1). The follow-up duration ranged from 12 to 58 months, with a mean time of (31 ± 17.12) months. One patient suffered from cranial surgery, and the pathological test revealed the IGM diagnosed. The other patients were with no recurrence.

## Discussion

**FIGURE 5:**
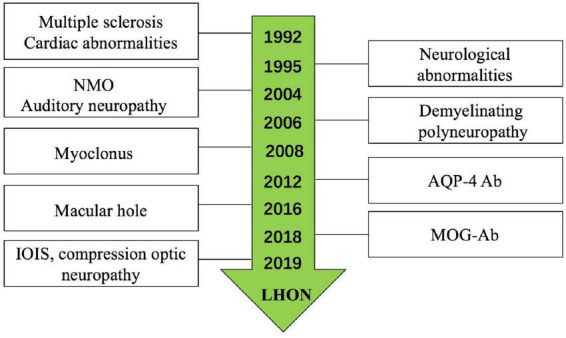
Leber’s hereditary optic neuropathy (LHON) plus had been reported for years.

Leber’s hereditary optic neuropathy plus has been reported by many researchers; a systematic review of the literature was conducted on all publications on LHON plus since the original description by Harding et al. (1992) (Figure 5; Bower et al., 1992; Harding et al., 1992; Nikoskelainen et al., 1995; Ceranić and Luxon, 2004; McFarland et al., 2007; La Morgia et al., 2008; Shiraishi et al., 2014; Shimada and Horiguchi, 2016; Bittner et al., 2019); the most common disease was LHON-MS (Harding disease) (Harding et al., 1992; Kellar-Wood et al., 1994; Bhatti and Newman, 1999). The last decade witnessed important discoveries in immune-mediated diseases of the optic nerve; the recognition of LHON plus is increasingly clear. There were studies reported LHON comorbidity with MOG-Ab (Bittner et al., 2019) and NMO (Bhatti and Newman, 1999). While in our single neuro-ophthalmology center, we found six (46.15%) patients who were comorbid with ON, in which two patients were with MOG-Ab positive (Shiraishi et al., 2014) and one patient with AQP4-Ab positive. The ratio of different subtypes of optic neuritis (ON) was higher than the other diseases; we analyzed the reason may be our center is the neuro-ophthalmology department, while the other centers are the neurology department. We also found LHON accompanied with IOIS and compression optic neuropathy, which were unreported before; only Jones et al. (2018) reported a case of saccular left renal artery aneurysm in a 27-year-old man with known LHON.

Approximately, 90% patients carry one of the three primary mtDNA mutations: 11778, 3460, or 14484 (Harding et al., 1995; Mackey et al., 1996; Newman and Biousse, 2004). While, in our study, the ratio of these three mutations was only 46.15% and none of the 14484 mutations were found. The clinical characteristics of LHON Plus may be varied. But the SD-OCT results show that more severe ganglion cell atrophy in the nasal quadrants is homogeneous, which reminds to test mtDNA mutations. The visual acuity of patients with LHON may be stable in a previous study; most patients may suffer low visual acuity (≤0.1). Although most patients with LHON Plus suffer severe vision loss, they will recover after accepted corrected and symptomatic treatment. According to the guideline, patients diagnosed with NMOSD should give immune modification therapy to involve relapse.

The most common disease in our study is CNS inflammatory demyelination disease; MRI is the modality of choice for investigating ON, compressive lesions, and the other diseases. An MRI can be performed to evaluate a patient’s potential benefit from intravenous methylprednisolone and disease-modifying therapy. Eleven patients presented with optic nerve or intracranial T2 lesions and (or) enhanced T1 lesions; nine patients received intravenous methylprednisolone therapy, two patients. So we suggest the patients who had diagnosed LHON should perform MRI examination.

A few limitations existed in this cohort study. First, we only report the ratio of LHON Plus in-patients in a single neuro-ophthalmology center. Second, the study only found LHON accompanied with AQP4-Ab positive, but the pathogeny is unknown; we cannot clearly explain the reason of the visual loss. Another limitation was the follow-up timer was not enough; we cannot make sure whether the patients were diagnosed with LHON or carried mtDNA mutations, especially in some patients who had one eye involved.

### In summary

Leber’s hereditary optic neuropathy plus may be a clinical syndrome associated with multiple diseases; even normal people will carry with mutations, which still need investigation. When field vision of the patient showed the blind spot in the temporal or SD-OCT imaging of the macular RGC-IPL revealed ganglion cell atrophy, more severe in the nasal quadrants; we suggested that the patient undergo LHON mutation examination. An MRI examination is necessary. The treatment should be dependent on the clinical and examination results; the prognosis is relatively ideal.

## Data availability statement

The original contributions presented in this study are included in the article/supplementary material, further inquiries can be directed to the corresponding authors.

## Ethics statement

Written informed consent was obtained from the individual(s), and minor(s)’ legal guardian/next of kin, for the publication of any potentially identifiable images or data included in this article.

## Author contributions

H-FZ and S-HW designed and conducted the study. M-MS, QS, H-EL, H-LS, H-JL, MY, and DT collected, analyzed, managed, and interpreted the data. M-MS and H-FZ prepared the manuscript and conducted the statistical analysis. H-FZ and Q-GX performed critical revision of the manuscript. All authors reviewed and approved the final version of the manuscript.
